# Supplementary figures and images for: Post-translational modification patterns on β-myosin heavy chain are altered in ischemic and nonischemic human hearts
Source: eLife. 2022 May 3;11:e74919. doi: 10.7554/eLife.74919 (PMC9122498; doi:10.7554/eLife.74919)

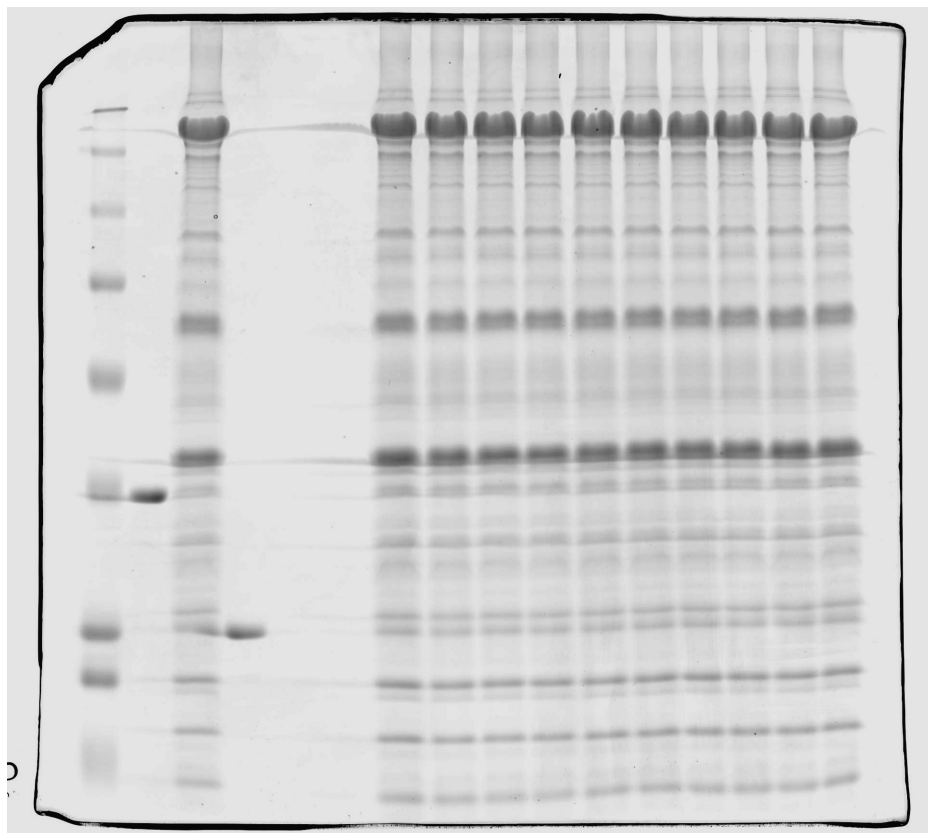

Supplement: Figure 1—figure supplement 1—source data 1. [file elife-74919-fig1-figsupp1-data1.pdf]
