# Supplementary material for: Post-translational modification patterns on β-myosin heavy chain are altered in ischemic and nonischemic human hearts
Source: eLife. 2022 May 3;11:e74919. doi: 10.7554/eLife.74919 (PMC9122498; doi:10.7554/eLife.74919)
Supplement: Supplementary file 1. — Deidentified human heart samples were obtained from nonfailing, ischemic heart failure, and nonischemic heart failure patients. [file elife-74919-supp1.docx]

**Summary of the patients’ demographic features**

| Sample # | | Failing Category | Age | | Gender | | Race |
| --- | --- | --- | --- | --- | --- | --- | --- |
| 1 | Non-failing | | 51 | F | | White | |
| 2 | Non-failing | | 69 | M | | White | |
| 3 | Non-failing | | 41 | M | | Black | |
| 4 | Non-failing | | 58 | M | | White | |
| 5 | Ischemic heart failure | | 50 | F | | White | |
| 6 | Ischemic heart failure | | 68 | M | | White | |
| 7 | Ischemic heart failure | | 47 | M | | Black | |
| 8 | Ischemic heart failure | | 62 | M | | Black | |
| 9 | Non-ischemic heart failure | | 53 | F | | Black | |
| 10 | Non-ischemic heart failure | | 68 | M | | White | |
| 11 | Non-ischemic heart failure | | 47 | M | | White | |
| 12 | Non-ischemic heart failure | | 61 | M | | | Black |

**Landim-Vieira et al. 2022**

**Supplemental Table I**
