# Supplementary material for: Post-translational modification patterns on β-myosin heavy chain are altered in ischemic and nonischemic human hearts
Source: eLife. 2022 May 3;11:e74919. doi: 10.7554/eLife.74919 (PMC9122498; doi:10.7554/eLife.74919)
Supplement: Supplementary file 2. — List of potential pathogenicity of the variants and their location within nearby PTM regions. Cardiomyopathy-loop (CM-loop), likely pathogenic (LP), pathogenic (P), hypertrophic cardiomyopathy (HCM), and dilated cardiomyopathy (DCM). [file elife-74919-supp2.docx]

| Modified Residue | | Nearby Variant | | Location | | Association by Literature | | Cardiomyopathy Designation | | Literature Review | | ClinVar (Classification) | |
| --- | --- | --- | --- | --- | --- | --- | --- | --- | --- | --- | --- | --- | --- |
| K34/ K58 | | R17C | | SH3 | | LP | | HCM | | Alamo et al. 2017 | | --------- | |
|  |  | A26V | | SH3 | | P | | HCM | | Van Driest et al. 2004 | | Benign | |
|  |  | V39M | | SH3 | | P | | HCM | | Van Driest et al. 2004 | | Conflicting interpretations of pathogenicity | |
|  |  | R54X | | SH3 | | P | | HCM | | Van Driest et al. 2004 | | --------- | |
|  |  | V59I | | SH3 | | P | | HCM | | Alamo et al. 2017 | | --------- | |
| G181R | | P-Loop | | P/LP | | DCM | | Alamo et al. 2017 | | --------- | |  |  |
| R190T | | Near P-Loop | | P | | HCM | | Van Driest et al. 2004 | | --------- | |  |  |
| Q193R | | Near P-Loop | | LP | | HCM | | Alamo et al. 2017 | | --------- | |  |  |
| A196T | | Near Loop 1 | | P | | HCM | | Woo et al. 2003 | | --------- | |  |  |
| A199V | | Near Loop 1 | | LP | | HCM | | Alamo et al. 2017 | | --------- | |  |  |
| I201T | | Near 25/50 junction (Loop1) | | P | | DCM | | Uniprot | | Pathogenic/ Likely pathogenic | |  |  |
| R204C | | Loop 1 | | P | | HCM | | Alamo et al. 2017 | | --------- | |  |  |
| R204H | | Loop 1 | | P | | HCM | | Uniprot | | Conflicting interpretations of Pathogenicity | |  |  |
| K207Q | | Loop 1 | | P | | HCM | | Uniprot | | Conflicting interpretations of pathogenicity | |  |  |
| P211L | | Loop 1 | | LP | | HCM | | Woo et al. 2003 | | Uncertain significance | |  |  |
| G214D | | Loop 1 | | P | | HCM | | Alamo et al. 2017 | | --------- | |  |  |
| Q222K | | Near Loop 1 | | P | | HCM | | Van Driest et al. 2004 | | --------- | |  |  |
| A223T | | Near Loop 1 | | P | | DCM | | Uniprot | | Uncertain significance | |  |  |
| L227V | | Near Switch 1 | | P | | HCM | | Uniprot | | --------- | |  |  |
| N232S | | Near Switch 1 | | Benign | | ---- | | Marin-Garcia et al. 2014 | | --------- | |  |  |
| N232H | | Near Switch 1 | | P | | HCM | | Alamo et al. 2017 | | --------- | |  |  |
| D239N | | Switch 1 | | LP | | HCM | | Alamo et al. 2017 | | --------- | |  |  |
| R243H | | Switch 1 | | P | | DCM | | Alamo et al. 2017 | | Conflicting interpretations of Pathogenicity | |  |  |
| G245E | | Switch 1 | | P | | DCM | | Alamo et al. 2017 | | --------- | |  |  |
| I248F | | Near Switch 1 | | p | | DCM | | Alamo et al. 2017 | |  | |  |  |
| K429 | | R403W/G/L/Q | | CM-Loop | | P | | HCM | | Alamo et al. 2017 | | Uncertain significance/ Pathogenic | |
|  |  | V406M | | CM-Loop | | LP | | HCM | | Alamo et al. 2017 | | --------- | |
|  |  | G407V | | CM-Loop | | LP | | HCM | | Uniprot | | Likely Pathogenic | |
|  |  | Y410D | | CM-Loop | | LP | | HCM | | Alamo et al. 2017 | | --------- | |
|  |  | V411I | | CM-Loop | | P | | HCM | | Woo et al. 2003 | | Conflicting interpretations of pathogenicity | |
|  |  | T412N | | CM-Loop | | P | | DCM | | Villard et al. 2005 | | --------- | |
|  |  | G425R | | Near CM-Loop | | P | | HCM | | Uniprot | | Conflicting interpretations of pathogenicity | |
|  |  | L427M | | Near CM-Loop | | LP | | HCM | | Alamo et al. 2017 | | --------- | |
|  |  | A428V | | Near CM-Loop | | P | | HCM | | Van Driest et al. 2004 | | Likely pathogenic | |
|  |  | A430E | | Near CM-Loop | | P | | HCM | | Uniprot | | Likely pathogenic; Uncertain significance. | |
|  |  | M435T | | Near Switch 2 | | P | | HCM | | Uniprot | | --------- | |
|  |  | V440M | | Upper 50 KDa Domain | | P | | HCM | | Van Driest et al. 2004 | | Conflicting interpretations of pathogenicity | |
|  |  | T441M | | Upper 50 KDa Domain | | Benign | | Myopathy | | Uniprot | | Conflicting interpretations of pathogenicity | |
|  |  | R442C | | Upper 50 KDa Domain | | P | | HCM | | Alamo et al. 2017 | | --------- | |
|  |  | I443T | | Near Switch 2 | | P | | HCM | | Van Driest et al. 2004 | | --------- | |
|  |  | T449S | | Near Switch 2 | | LP | | HCM | | Alamo et al. 2017 | | --------- | |
|  |  | R453C | | Sequentially Near Switch 2 | | P | | HCM | | Alamo et al. 2017 | | Uncertain significance/ Pathogenic | |
|  |  | R453S/H | | Sequentially Near Switch 2 | | LP | | HCM | | Alamo et al. 2017 | | Uncertain significance/ Pathogenic | |
| K951 | | E930K | | Ring 2 | | P | | HCM | | Alamo et al. 2017 | | Pathogenic/Likely Pathogenic | |
|  |  | E931K | | Ring 2 | | P | | HCM | | Van Driest et al. 2004 | | --------- | |
|  |  | E935K | | Ring 2 | | P | | HCM | | Van Driest et al. 2004 | | Pathogenic | |
|  |  | E949K | | Ring 3 | | P | | HCM | | Anan et al. 1994 | | Uncertain significance | |
|  |  | D953H | | Ring 3 | | P | | HCM | | Van Driest et al. 2004 | | --------- | |
|  |  | L961R | | Near Ring 3 | | P | | HCM | | Van Driest et al. 2004 | | --------- | |
|  |  | E967K | | Near Ring 3 | | LP | | HCM | | Alamo et al. 2017 | | --------- | |
| K1195 | | R1193S | | Near Skip 1 | | P | | DCM | | Villard et al 2005 | | Uncertain significance | |
|  |  | R1193H | | Near Skip 1 | | P | | DCM | | Alamo et al. 2017 | | --------- | |

**Landim-Vieira et al. 2022**

**Supplemental Table 2**
