# Supplementary material for: Post-translational modification patterns on β-myosin heavy chain are altered in ischemic and nonischemic human hearts
Source: eLife. 2022 May 3;11:e74919. doi: 10.7554/eLife.74919 (PMC9122498; doi:10.7554/eLife.74919)
Supplement: Transparent reporting form [file elife-74919-transrepform1.pdf]

Westbrook Centre
Milton Road
Cambridge CB4 1YG, UK
P 01223 855340
W elifesciences.org
T @elife

eLife's transparent reporting form

We encourage authors to provide detailed information *within their submission* to facilitate the interpretation and replication of experiments. Authors can upload supporting documentation to indicate the use of appropriate reporting guidelines for health-related research (see EQUATOR Network), life science research (see the BioSharing Information Resource), or the ARRIVE guidelines for reporting work involving animal research. Where applicable, authors should refer to any relevant reporting standards documents in this form.

If you have any questions, please consult our Journal Policies and/or contact us: editorial@elifesciences.org.

Sample-size estimation

- You should state whether an appropriate sample size was computed when the study was being designed
- You should state the statistical method of sample size computation and any required assumptions
- If no explicit power analysis was used, you should describe how you decided what sample (replicate) size (number) to use

Please outline where this information can be found within the submission (e.g., sections or figure legends), or explain why this information doesn't apply to your submission:

The sample size of human hearts was determined by the number available. The statistical methods utilized is reported in the Methods Section under the heading Statistical Analysis. No explicit power analysis was used, a larger number of samples may have increased the power of comparisons in certain cases.

Replicates

- You should report how often each experiment was performed
- You should include a definition of biological versus technical replication
- The data obtained should be provided and sufficient information should be provided to indicate the number of independent biological and/or technical replicates
- If you encountered any outliers, you should describe how these were handled
- Criteria for exclusion/inclusion of data should be clearly stated
- High-throughput sequence data should be uploaded before submission, with a private link for reviewers provided (these are available from both GEO and ArrayExpress)

Please outline where this information can be found within the submission (e.g., sections or figure legends), or explain why this information doesn't apply to your submission:

LC-mass spectroscopy was performed for each of the 12 heart samples obtained, 4 samples each for non-failing, ischemic heart failure and non-ischemic heart failure. These were run as biological replicates, all of the sample was utilized, therefore no technical replicates were performed. Several outliers were encountered, they were left in the data sets and not removed as specified under the Statistical Analysis section. The mass spec data was uploaded to Dyrad using the following link: https://datadryad.org/stash/share/SFRwIAR8tdqmV2PQXayHoD363NCfbgezTXZpJCqzMTc. The dataset was assigned a unique identifier, DOI (doi:10.5061/dryad.s4mw6m97g), which has been added to the Methods Section under the Liquid Chromatography-Mass Spectrometry heading. Files containing molecular dynamics information were provided under source information, 3 runs were performed per simulation.

Statistical reporting

- Statistical analysis methods should be described and justified
- Raw data should be presented in figures whenever informative to do so (typically when N per group is less than 10)
- For each experiment, you should identify the statistical tests used, exact values of N, definitions of center, methods of multiple test correction, and dispersion and precision measures (e.g., mean, median, SD, SEM, confidence intervals; and, for the major substantive results, a measure of effect size (e.g., Pearson's r, Cohen's d)
- Report exact p-values wherever possible alongside the summary statistics and 95% confidence intervals. These should be reported for all key questions and not only when the p-value is less than 0.05.

Please outline where this information can be found within the submission (e.g., sections or figure legends), or explain why this information doesn't apply to your submission:

> Statistical analysis methods are listed under Methods section but also the exact N values and mean $\pm$ SD were listed in the figure legends where statistical analysis had been performed. The Supplemental Tables 3 and 4 contain information on N.

(For large datasets, or papers with a very large number of statistical tests, you may upload a single table file with tests, Ns, etc., with reference to sections in the manuscript.)

Group allocation

- Indicate how samples were allocated into experimental groups (in the case of clinical studies, please specify allocation to treatment method); if randomization was used, please also state if restricted randomization was applied
- Indicate if masking was used during group allocation, data collection and/or data analysis

Please outline where this information can be found within the submission (e.g., sections or figure legends), or explain why this information doesn't apply to your submission:

> The mass spec samples were masked during data acquisition and unmasked for analysis.

Additional data files ("source data")

- We encourage you to upload relevant additional data files, such as numerical data that are represented as a graph in a figure, or as a summary table
- Where provided, these should be in the most useful format, and they can be uploaded as "Source data" files linked to a main figure or table
- Include model definition files including the full list of parameters used
- Include code used for data analysis (e.g., R, MatLab)
- Avoid stating that data files are "available upon request"

Please indicate the figures or tables for which source data files have been provided:

> Source data has been provided for Figures 2, 3, 7, 8 , 9 and Supplemental Figure 1, 2, 4, Tables 1, 2, 3 and 4.

eLife Sciences Publications, Ltd is a limited liability non-profit non-stock corporation incorporated in the State of Delaware, USA, with company number 5030732, and is registered in the UK with company number FC030576 and branch number BR015634 at the address Westbrook Centre, Milton Road, Cambridge CB4 1YG.
